# Recent Emergence of *Staphylococcus aureus* Clonal Complex 398 in Human Blood Cultures

**DOI:** 10.1371/journal.pone.0041855

**Published:** 2012-10-18

**Authors:** Erwin Verkade, Anneke M. C. Bergmans, Andries E. Budding, Alex van Belkum, Paul Savelkoul, Anton G. Buiting, Jan Kluytmans

**Affiliations:** 1 Laboratory for Microbiology and Infection Control, Amphia Hospital, Breda, The Netherlands; 2 Laboratory for Medical Microbiology and Immunology, St. Elisabeth Hospital, Tilburg, The Netherlands; 3 Laboratory for Microbiology, Franciscus Hospital, Roosendaal, The Netherlands; 4 Department of Medical Microbiology, VU University medical centre, Amsterdam, The Netherlands; 5 Department of Medical Microbiology and Infectious Diseases, Erasmus Medical Center, Rotterdam, The Netherlands; 6 bioMérieux, Microbiology R and D, La Balme Les Grottes, France; University of Iowa, United States of America

## Abstract

**Background:**

Recently, a clone of MRSA with clonal complex 398 (CC398) has emerged that is related to an extensive reservoir in animals, especially pigs and veal calves. It has been reported previously that methicillin-susceptible variants of CC398 circulate among humans at low frequency, and these have been isolated in a few cases of bloodstream infections (BSI). The purpose of this study was to determine the prevalence of *S. aureus* CC398 in blood cultures taken from patients in a geographic area with a high density of pigs.

**Methodology/Principal Findings:**

In total, 612 consecutive episodes of *S. aureus* BSI diagnosed before and during the emergence of CC398 were included. Three strains (2 MSSA and 1 MRSA) that were isolated from bacteremic patients between 2010–2011 were positive in a CC398 specific PCR. There was a marked increase in prevalence of *S. aureus* CC398 BSI isolated between 2010–2011 compared to the combined collections that were isolated between 1996–1998 and 2002–2005 (3/157, 1.9% *vs.* 0/455, 0.0%; *p* = 0.017).

**Conclusions/Significance:**

In conclusion, in an area with a relative high density of pigs, *S. aureus* CC398 was found as a cause of BSI in humans only recently. This indicates that *S. aureus* CC398 is able to cause invasive infections in humans and that the prevalence is rising. Careful monitoring of the evolution and epidemiology of *S. aureus* CC398 in animals and humans is therefore important.

## Introduction

Traditionally, methicillin-resistant *Staphylococcus aureus* (MRSA) has been considered a hospital associated pathogen. Since the end of the last century, MRSA has expanded its territory to the community causing severe infections in previously healthy persons all over the world [1]–[2]. In 2003 a new clone of MRSA was first identified that was related to an extensive reservoir in pigs and cattle [3]. People who are in direct contact with pigs and veal calves have high carriage rates of such MRSA (23% and 29%, respectively) [3]–[4]. The livestock-associated MRSA CC398 strains are characterized by being non-typeable by pulsed-field gel electrophoresis (PFGE) and are, therefore, sometimes referred to as non-typeable MRSA (NT-MRSA) [5]. Using multi locus sequence typing (MLST) these strains all belong to the sequence type 398. Remarkably, *S. aureus* CC398 harbors several staphylococcal cassette chromosome *mec* (SCC*mec*) types, suggesting that the resistance cassette was acquired on multiple unrelated occasions [5].

Livestock is the first clearly defined non-human reservoir of MRSA in the community. In the Netherlands MRSA CC398 was first detected in 2003, and by the end of 2007 nearly 30% of all newly identified MRSA in humans in the Netherlands were of this type, suggesting a recent and very rapid spread [6]. MRSA CC398 is now considered a zoonotic pathogen, affecting mainly people who work with pigs and veal calves. If this strain can spread successfully from human to human and also cause diseases in healthy individuals it might constitute a significant public health problem in the near future.

A typing method for *S. aureus*, based upon length polymorphism of the 16S–23S spacer region has been described previously. Although shown to be effective and reproducible [7], this method has not been widely implemented. It is based upon the fact that every *S. aureus* strain has 5 or 6 spacer regions in its chromosome. The length of the individual regions varies within the chromosome, so when amplified and sorted by length using agarose gel electrophoresis, each strain produces an individual pattern of bands. Typing based on these spacer patterns has been shown to correlate well with traditional typing methods [8], with discriminatory power comparable to that of MLST [9]–[10]. To confirm the results of the 16S–23S spacer fingerprint analysis, we additionally performed two specific polymerase chain reactions (PCR) for the detection of *S. aureus* CC398 isolates as described previously [11].

Recently, van Belkum and colleagues reported that methicillin-susceptible *S. aureus* (MSSA) isolates homologous to the MRSA CC398 were found among isolates derived from bacteremic patients (3 [2.1%] of 146) [12]. However, this study was based on a strain collection obtained from inhabitants of a densely populated, urban area. Moreover, the strains were not from consecutive episodes of bloodstream infections (BSI), which may have caused selection bias. The purpose of the current study was to determine the prevalence of *S. aureus* CC398 in consecutive BSI episodes from patients in an area with a high density of pigs.

## Materials and Methods

### Ethics Statement

Medical ethics review was not required for this study according to the Dutch Medical Research Involving Human Subjects Act (article 1, paragraph 1, section b of the WMO) due to the fact that patients were not physically involved in this study. In addition, privacy of patients was provided by coding all tested isolates according to the requirements of the National Privacy Regulations in the Netherlands and thus waived the need for consent. All blood cultures were taken routinely from patients with body temperatures higher than 39°C and symptoms of BSI for screening of micro-organisms. Isolates of the St. Elisabeth Hospital and Amphia Hospital were kindly provided by Anton Buiting and Jan Kluytmans, respectively.

### Strain Collections

Three independent collections with a total of 612 *S. aureus* isolates from patients with BSI were tested. The patients were hospitalized in one of two hospitals (St. Elisabeth Hospital, Tilburg or Amphia Hospital, Breda, the Netherlands). The cities of Tilburg and Breda are located in the southeast of the Netherlands with a high density of pigs, i.e. approximately 1,000 pigs per square kilometer [13]. The first collection consisted of 250 *S. aureus* strains that were isolated from consecutive episodes of BSI that occurred between January 1996 and February 1998, before MRSA CC398 had been reported. The second collection consisted of 205 *S. aureus* strains that were isolated from consecutive episodes of BSI that occurred between August 2002 and August 2005, when MRSA CC398 emerged in the Netherlands. The third collection comprised 157 *S. aureus* isolates that were isolated from consecutive episodes of BSI that occurred between January 2010 and April 2011, when MRSA CC398 was the most frequently observed MRSA variant in the hospitals involved. Only one isolate was included per patient per bacteremic episode. All isolates were identified by a latex agglutination test (Staphaurex Plus; Murex Diagnostics Ltd., Dartford, England), and by the detection of DNase (DNase agar; Oxoid Ltd., Basingstoke, England). The blood cultures isolates were classified as methicillin-susceptible (MIC of oxacillin ≤2 µg/ml) at the time of collection by broth micro-dilution susceptibility testing performed according to CLSI standards [14]. Resistance profiles to 21 antimicrobial agents were determined with the VITEK system (bioMérieux SA, Craponne, France) for all confirmed *S. aureus* strains according to the manufacturer's instructions.

### Samples and Microbiological Procedures

The isolates were stored at −80°C in Microbank™ (Pro-Lab Diagnostics) preservation system until they were tested. All strains were cultured overnight at 35°C on Columbia agar plates with 5% sheep blood to obtain fresh growth. A suspension with an optical density of 1.0 McFarland was made in 2 ml of 0.75% NaCl. From this suspension 500 µl was centrifuged at 14,000 rpm for 3 minutes in an Eppendorf centrifuge. The supernatant was removed and the pellet was resuspended in 200 µl distilled water (Baxter Healthcare SA, Zurich, Switzerland) by vortexing. This hypotonic fluid caused sufficient lysis of *S. aureus*. Subsequently, the suspension was centrifuged at 14,000 rpm for 3 minutes. The supernatant was used for PCR amplification without further processing.

### 16S–23S spacer fingerprint analysis

For amplification of the 16S–23S rDNA spacer regions, two primers were constructed in conserved regions of the 16S and 23S rRNA genes, as described previously [7]. Each PCR mixture with a final volume of 15 µl contained, 1 µl of DNA, 1× SuperTaq Buffer (HT Biotechnology Ltd, Cambridge, England), 0.25 U of SuperTaq polymerase (HT Biotechnology Ltd), 1.5 mM MgCl2, 67 µM deoxynucleotide triphosphates (HT Biotechnology Ltd), 0.5 µl of 1.25% bovine serum albumin and 1 µM of each primer [15]. The amplification was carried out on a 7500 Real-Time PCR System (Applied Biosystems, Foster City, USA). Cycling conditions for PCR were: 72°C for 2 min, 35 cycles of 94°C for 30 sec, 56°C for 45 sec, 72°C for 1 min and a final extension at 72°C for 10 min.

Subsequently, 7 µl of PCR product was mixed with 5 µl of loading buffer and separated on a 2.0% agarose gel. Electrophoresis was performed at 300 V during 90 minutes. Fragments lengths were sized using a 100 bp ladder DNA marker (Invitrogen, Carlsrad, USA). Next, band patterns were visualized using a UV Transilluminator (BioDoc-It System UVP, Upland, USA). Total time needed from cultured strains to spacer fingerprints was about 4 hours. This typing method for *S. aureus* has recently been validated using an extensive collection of strains and could detect all non-typeable MRSA in that collection [15]. The MRSA CC398 control strain used in our assay was isolated from a pig farmer and was non-typeable with PFGE using the restriction enzyme *Sma*I. This strain had a *spa*-type t108 and contained the SCC*mec* type V. Discrimination of MRSA CC398 from other *S. aureus* strains using spacer fingerprints is easy due to the detection of the 539 bp fragment that is unique to MRSA CC398 strains. This method was only applied on the strains isolated before 2007.

### CC398 specific PCR

The CC398 specific primers C01F and C01R were used as described previously in order to detect *S. aureus* CC398 strains by PCR [10]. In addition, a CC398 specific probe C01P-FAM (FAM-GTCAGTATGAATTGCGGTATG-BHQ1) was constructed to visualize DNA amplification by real-time PCR. For amplification of *S. aureus* sequence, the primers A04F and A04R were used as described previously to check all tested strains for amplifiable *S. aureus* DNA [10]. Furthermore, probe A04P-YY (Yakima Yellow-GAGATTTGAGTTTGTGATACACCTGA-BHQ1) was constructed to visualize DNA amplification in real-time. Each PCR mixture with a final volume of 25 µl contained, 2 µl of DNA and 12.5 µl of TaqMan® Fast Universal PCR Master Mix (Applied Biosystems, Foster City, USA). For the CC398 specific PCR, concentrations of 600 nM of primer C01F, 600 nM of primer C01R and 150 nM of probe C01P-FAM were used. For the *S. aureus* specific PCR, concentrations of 900 nM of primer A04F, 400 nM of primer A04R and 100 nM of probe A04P-YY were used. The amplification was carried out on a 7500 Real-Time PCR System (Applied Biosystems, Foster City, USA). PCRs were performed using the following cycling protocol: 95°C for 10 min, followed by 35 cycles of 95°C for 15 sec, 58°C (C01 primers) or 54°C (A04 primers) for 30 sec, 60°C for 1 min. All 612 *S. aureus* strains were analyzed with the CC398 specific PCR.

### Genotyping of S. aureus CC398 strains. S. aureus

CC398 strains were genotyped by staphylococcal protein A (spa) typing [16].

## Results

In total, 612 *S. aureus* BSI were diagnosed in 367 males and 245 females. The vast majority were methicillin-susceptible (MSSA), 610 cases (99.7%) versus 2 cases of MRSA (0.3%). The MRSA cases were both from the most recent collection. The strains from the first and second collection were typed using the 16S–23S spacer fingerprint analysis, which showed that none of the 455 *S. aureus* strains had a fingerprint that corresponded to the MRSA CC398. The spacer fingerprints were digitalized and compared using the BioNumerics software package (Applied Maths, Sint-Martens-Latem, Belgium). Typically, MRSA CC398 strains were characterized by the presence of fragments of 369, 386, 399, 520, 539 and 601 base pairs (bp) in length. The 539 bp fragment was encountered only in livestock-associated strains [15].

To confirm the results of the 16S–23S spacer fingerprint analysis, a CC398 specific PCR protocol was performed on all 612 *S. aureus* strains. All strains were positive in the *S. aureus* specific PCR, demonstrating that all 612 DNA samples used for PCR contained amplifiable *S. aureus* DNA. In addition, CC398 specific PCR analysis demonstrated that 3 out of these 612 *S. aureus* isolates (0.5%) were positive using the protocol. Prevalence was the highest among MRSA (1/2, 50% *vs.* 2/610, 0.3% for MSSA; *p* = 0.010). The three *S. aureus* CC398 strains (1 MRSA strain originated from Tilburg and 2 MSSA strains of which one originated from Tilburg and one from Breda) all belonged to the third collection which was obtained between January 2010 and April 2011.

The prevalence of *S. aureus* CC398 BSI increased recently. A significantly higher prevalence was found in the most recent collection compared with the older collections (3/157, 1.9% *vs.* 0/455, 0.0%; *p* = 0.017). We found only one MRSA isolate with the typical pig-borne *spa*-type t011. The MRSA strain was isolated in Tilburg in March 2010 and was resistant to tetracycline, trimethoprim/sulfadiazine and gentamicin. Examination of the patient history revealed no known contact with livestock. The patient was a 72-year-old woman with an infected shoulder prosthesis. The two MSSA CC398 isolates found in our study had both *spa*-type t571 and were only resistant to erythromycin. These two cases were hospital-acquired and included 1 case of catheter-associated bloodstream infection observed in a 71-year-old woman with complicated perforation of the intestines (isolated in Breda in February 2011), and 1 case following cholecystitis in a 84-year-old woman (recovered in Tilburg in November 2010). None of the three *S. aureus* CC398 isolates were positive for the Panton-Valentine Leukocidin (PVL) gene. The mode of acquisition of the *S. aureus* CC398 isolates by our 3 patients remains unclear.

To ensure that both protocols used in this study can detect *S. aureus* CC398, five of the six MSSA CC398 strains found in the study of van Belkum et al. [12] were analyzed using the 16S–23S spacer fingerprint analysis protocol and the CC398 specific PCR. Both strains isolated from the nose swabs and the three strains isolated from blood cultures showed a 16S–23S spacer fingerprint that corresponded to our MRSA CC398 control strain (data not shown). Moreover, these five strains as well as our positive control strain were also positive in both the *S. aureus* specific and the CC398 specific PCR.

## Discussion

A large collection of consecutive isolates of *S. aureus* BSI episodes in an area with a high density of pigs revealed the presence of *S. aureus* CC398 in blood cultures. We demonstrated that three of the 612 *S. aureus* strains isolated from bacteremic patients living in an area with a high density of pigs belong to CC398. These three strains were all isolated in 2010–2011. Conversely, none of the 455 strains that were isolated in 1996–1998 and 2002–2005 belong to CC398. Therefore, the present study suggests a recent emergence among Dutch patients of BSI with CC398, a *Staphylococcus aureus* strain formerly found primarily in association with pigs [12].

In accordance to our findings, van Belkum and colleagues [12] found that CC398 is rare among Dutch MSSA strains colonizing healthy persons (2 [0.2%] of 829 strains). However, a higher number of MSSA isolates belonging to clonal complex 398 were found among bacteremic patients (3 [2.1%] of 146; *p* = 0.026). The prevalence of MSSA CC398 in human blood cultures was comparative with the prevalence found in our study. Interestingly, all three *S. aureus* CC398 strains found by van Belkum and colleagues were isolated from bacteremic patients in Rotterdam had also *spa*-type t571. The authors were unable to find an epidemiological link between these 3 patients. The strains were obtained from different patients in different medical departments over an extended period. Notably, *spa*-type t571 CC398 has been found recently in MSSA carriage isolates from New York City [17], and the Dominican Republic [17], and also in clinical MSSA isolates from northern Manhattan [18], Colombia [19], the Netherlands [12], and France [20]. A recent study even postulates that MRSA CC398 originated in human as MSSA, with *spa*-type t571 disproportionately common among human-associated isolates [21]. The lineage appears to have undergone a rapid radiation in conjunction with the jump from humans to livestock. This jump was accompanied by the loss of phage-carried human virulence genes, which likely attenuated its zoonotic potential [21].

Another recently published study demonstrated that the incidence of *S. aureus* CC398 BSI is rapidly increasing. Valentin-Domelier et al. reported 18 *S. aureus* CC398 BSI during a four-year period in three noncontiguous French regions [22]. *S. aureus* CC398 incidence showed a seven-fold increase during the study period (0.002 per 1,000 patient days in 2007 *vs.* 0.014 in 2010). Seventeen out of the 18 BSI isolates were methicillin-susceptible and additional *spa*-typing identified six related *spa*-types. The two *spa*-types t571 and t1451, that are infrequently associated with pig-borne isolates, were found with 10 and 4 BSI isolates, respectively. No isolate was assigned to classical pig-borne *spa*-types, such as t011, t034 or t108. None of the 18 CC398 isolates were positive for the PVL gene.

At present, it is unclear whether MRSA CC398 is easily transmissible from human to human. However, recent published studies demonstrate that MRSA CC398 is less transmissible than other MRSA strains [23]–[24]. In contrast, a recent study reported that CC398 is a frequent source of MSSA infections in northern Manhattan and is readily transmitted between individuals in households [18]. Also the virulence of *S. aureus* CC398 is still a matter of debate. There are indications that MRSA CC398 causes infections less frequently than other MRSA strains [24]–[25]. On the other hand, there are increasing numbers of case reports of severe invasive infections due to MRSA CC398, such as endocarditis and necrotizing pneumonia [26]–[27]. In addition, these recent invasive infections are mostly associated with non-pig-borne isolates [11], [20], [22]. To determine whether the potential impact of MRSA CC398 can be derived from the existence of genetically homologous MSSA strains among bacteremic patients, we determined the prevalence of *S. aureus* CC398 in positive blood cultures taken from patients in an area with a high prevalence of pigs using 16S–23S spacer fingerprint analysis and also a CC398 specific PCR. The historical occurrence of *S. aureus* CC398 in blood culture isolates may provide an estimate for the potential impact of MRSA CC398 on public health.

The results of our study have two important limitations. Firstly, approximately one third of the *S. aureus* BSI of the earlier period 1996–1998 were community-associated. The proportion of community-associated BSI increased to 50% in the period 2002–2005 and 2010–2011, respectively. One might expect that MRSA CC398 would be found more frequently in community-associated cases as compared to healthcare-associated cases based on the epidemiology known to date. Therefore, the likelihood to demonstrate the presence of *S. aureus* CC398 in blood culture may be reduced by including more healthcare-associated cases. Secondly, the first collection of strains was isolated in 1996–1998. This may have been too early as the first case of MRSA CC398 was reported only in 2003. It is possible that *S. aureus* CC398 was not present in livestock as well as humans in the Netherlands at that time. Considering the findings of Armand-Lefevre et al. this possibility is highly unlikely [28]. They found MSSA CC398 strains that were isolated from swine infections and also from healthy pig farmers between 1996–2002. MSSA CC398 was retrieved from 6 out of the 44 (13.6%) studied isolates from healthy pig farmers. In addition, four of the 14 (28.6%) isolates which were derived from a swine infection were MSSA CC398. The second collections of strains were isolated between 2002–2005 and 2010–2011 when MRSA CC398 was present in our country.

In conclusion, the rapid emergence of MRSA CC398 in the Netherlands is worrying [3]. The first isolate was found in 2003, and by the end of 2008 nearly 42% of all newly identified MRSA in humans reported to the National Institute of Public Health and the Environment in the Netherlands were MRSA CC398 (www.rivm.nl/mrsa). Approximately one third of people who are in direct contact with pigs and veal calves carry MRSA [29] and there is no indication that the extensive reservoir in animals can be brought under control in the near future. *S. aureus* CC398 is a clonal complex that has been associated with pigs and pig farmers before [28]. Our results indicated that *S. aureus* CC398 might be an increasing cause of invasive staphylococcal disease. These results are in line with other findings that certain *S. aureus* CC398 isolates, especially *spa*-type t571, can cause invasive infections in humans [12], [18], [21], [22], [27]. This human-adapted CC398 subclone is now increasingly identified in hospitals [20]. It appears to be highly receptive for horizontal gene transfer [30]. Therefore, further adaptation to humans may occur and if *S. aureus* CC398 can successfully spread from human to human it may pose a significant public health problem in the future. In addition, further acquisition of genetic elements harboring virulence and antibiotic resistance could arise. Therefore, careful monitoring of the evolution and epidemiology of MRSA CC398 is important.
